# Mining Sensor Data to Assess Changes in Physical Activity Behaviors in Health Interventions: Systematic Review

**DOI:** 10.2196/41153

**Published:** 2023-03-06

**Authors:** Claudio Diaz, Corinne Caillaud, Kalina Yacef

**Affiliations:** 1 School of Computer Science The University of Sydney Sydney Australia; 2 Charles Perkins Centre School of Medical Sciences The University of Sydney Sydney Australia

**Keywords:** activity tracker, wearable electronic devices, fitness trackers, data mining, artificial intelligence, health, education, behavior change, physical activity, wearable devices, trackers, health education, sensor data

## Abstract

**Background:**

Sensors are increasingly used in health interventions to unobtrusively and continuously capture participants’ physical activity in free-living conditions. The rich granularity of sensor data offers great potential for analyzing patterns and changes in physical activity behaviors. The use of specialized machine learning and data mining techniques to detect, extract, and analyze these patterns has increased, helping to better understand how participants’ physical activity evolves.

**Objective:**

The aim of this systematic review was to identify and present the various data mining techniques employed to analyze changes in physical activity behaviors from sensors-derived data in health education and health promotion intervention studies. We addressed two main research questions: (1) What are the current techniques used for mining physical activity sensor data to detect behavior changes in health education or health promotion contexts? (2) What are the challenges and opportunities in mining physical activity sensor data for detecting physical activity behavior changes?

**Methods:**

The systematic review was performed in May 2021 using the PRISMA (Preferred Reporting Items for Systematic Reviews and Meta-Analyses) guidelines. We queried the Association for Computing Machinery (ACM), IEEE Xplore, ProQuest, Scopus, Web of Science, Education Resources Information Center (ERIC), and Springer literature databases for peer-reviewed references related to wearable machine learning to detect physical activity changes in health education. A total of 4388 references were initially retrieved from the databases. After removing duplicates and screening titles and abstracts, 285 references were subjected to full-text review, resulting in 19 articles included for analysis.

**Results:**

All studies used accelerometers, sometimes in combination with another sensor (37%). Data were collected over a period ranging from 4 days to 1 year (median 10 weeks) from a cohort size ranging between 10 and 11615 (median 74). Data preprocessing was mainly carried out using proprietary software, generally resulting in step counts and time spent in physical activity aggregated predominantly at the daily or minute level. The main features used as input for the data mining models were descriptive statistics of the preprocessed data. The most common data mining methods were classifiers, clusters, and decision-making algorithms, and these focused on personalization (58%) and analysis of physical activity behaviors (42%).

**Conclusions:**

Mining sensor data offers great opportunities to analyze physical activity behavior changes, build models to better detect and interpret behavior changes, and allow for personalized feedback and support for participants, especially where larger sample sizes and longer recording times are available. Exploring different data aggregation levels can help detect subtle and sustained behavior changes. However, the literature suggests that there is still work remaining to improve the transparency, explicitness, and standardization of the data preprocessing and mining processes to establish best practices and make the detection methods easier to understand, scrutinize, and reproduce.

## Introduction

Wearable sensors are increasingly employed in health interventions because of their ability to track participants’ physical activity (PA) in an unobtrusive, continuous, and precise manner under free-living conditions [1]. In the context of health promotion, sensor data are commonly used to objectively assess interventions by monitoring PA changes and progress toward compliance with public health PA guidelines [2].

The rich data captured by activity sensors contain information about the participants’ PA, potentially unlocking valuable insights into PA behaviors and patterns [3]. These insights can help to advance the understanding of how interventions affect PA behaviors and how behaviors change, thereby scaffolding the design of future interventions, and enhancing their outcomes, efficacy, and adherence.

In the last decade, a growing number of artificial intelligence and data mining models and techniques have been developed to detect and extract these latent PA patterns beyond the typical summaries of pre- and postintervention daily steps or time spent in various PA levels. In this systematic review, we aimed to describe the data mining models and techniques currently used to detect PA with a focus on behavior changes. We discuss their value, identify gaps or challenges, and highlight opportunities. The following research questions (RQs) guided this review:

RQ1: What are the current techniques used for mining PA sensor data to detect behavior changes in health education or health promotion contexts?

RQ1.1 What are the types of sensors used and what data are collected?

RQ1.2 How are data preprocessed?

RQ1.3 What features are used to detect behavior changes?

RQ1.4 What are the data mining models and techniques used to detect behavior changes?

RQ1.5 What are the interpretation of data mining models used for?

RQ2: What are the challenges and opportunities in mining PA sensor data for detecting PA behavior changes?

**Figure 1:**
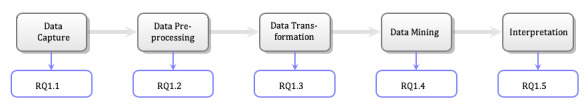
Knowledge discovery in database steps (in grey) and research question 1 (RQ1) subquestions (in blue).

The RQ1 subquestions were established following the reasoning and order of the process of knowledge discovery in databases [4]. Figure 1 summarizes this process and maps each step with the relevant RQ1 subquestion.

## Methods

### Design

For this systematic review, we followed the PRISMA (Preferred Reporting Items for Systematic Reviews and Meta-Analyses) guidelines [5] and used the Rayyan QCRI web application [6] to manage the review process. We identified studies by searching the Association for Computing Machinery (ACM), IEEE Xplore, ProQuest, Scopus, Web of Science, Education Resources Information Center (ERIC), and Springer digital libraries. We also searched Google Scholar to identify grey literature and extracted the first 100 results. For this scholarly reference search, we used the following query: (education OR promotion OR “behaviour change”) AND (“data mining” OR “machine learning” OR “artificial intelligence”) AND (sensor OR accelerometer OR tracker OR wearable) AND “physical activity” AND health. All extracted scholarly references had been added to the database at the latest on the search day (May 28, 2021). The inclusion and exclusion criteria are presented in Textbox 1.

**Inclusion and exclusion criteria for article selection in the review.**

**Inclusion criteria**

- Full-length articles
- Peer-reviewed articles in journals or conference papers
- Articles that used data mining techniques for data from physical activity (PA) wearable sensors
- Articles that included PA data
- Articles on applied health education/promotion or on behavior change scenarios
- Articles that used well-known data mining techniques such as classification, regression, clustering, association, and sequence algorithms, as well as specific algorithms to model PA data

**Exclusion criteria**

- Use of analytics without data mining
- Studies on animals (eg, accelerometers on dogs)
- Self-quantification without a health education or health motivation component
- Dissertations and theses, due to lack of a peer review process
- Systematic reviews, reviews, and meta-analyses
- Health care applications without a health education or motivation for behavior change component
- Specific movement detection (abnormal gait, falls)
- Aid for sport training (eg, maintaining heart rate, postures, specific movements)

### Search Outcome

The number of references extracted from each electronic database is summarized in Table 1.

**Figure 2:**
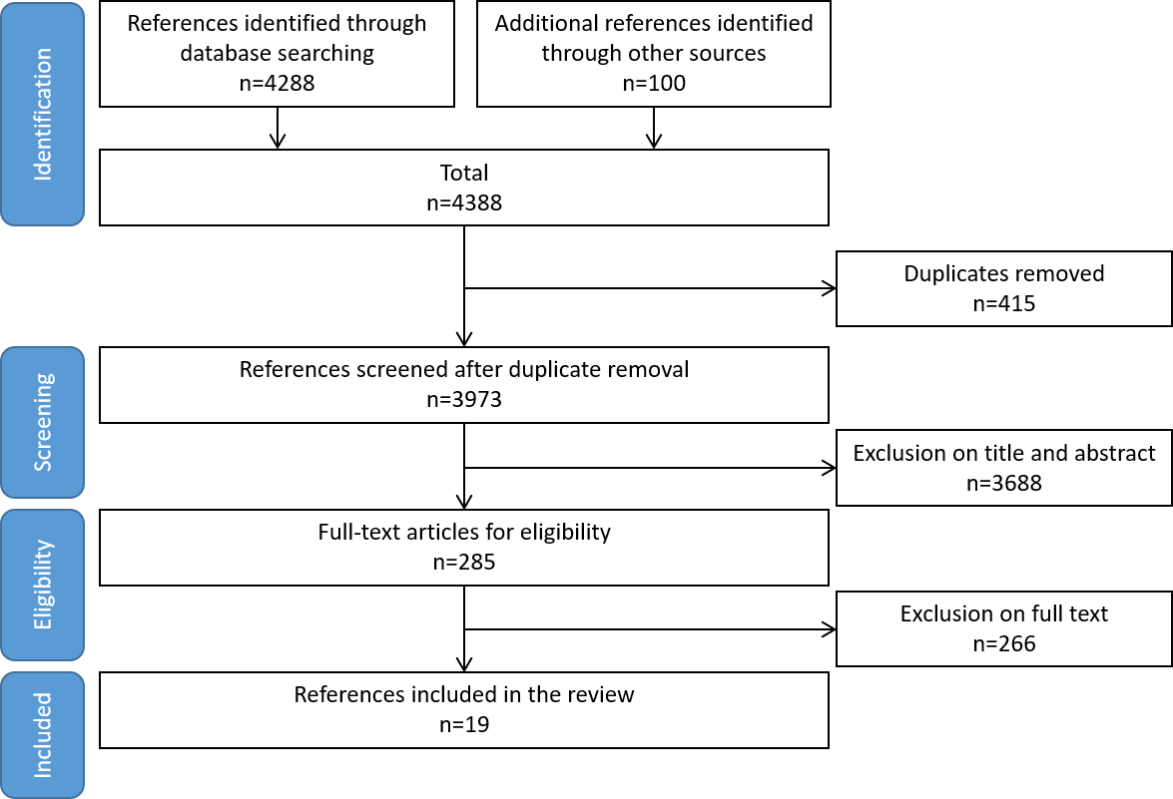
Study inclusion flowchart according to the PRISMA (Preferred Reporting Items for Systematic Reviews and Meta-Analyses) methodology.

Following the PRISMA methodology, we retrieved 4388 references from the sources listed in Table 1. We then removed 415 duplicates, leaving 3973 unique references that were screened by reading their titles and abstracts. Using the inclusion/exclusion criteria (Textbox 1), we excluded 3688 references and selected 285 publications. After full-text reading, we excluded 266 references: 33 on activity recognition, 5 on data mining, 24 on systems, 31 on rehabilitation, 39 not on behavior changes, 54 without data mining, 51 not on health education/promotion, 13 not on PA, and 16 reviews. At the end of the selection process (summarized in Figure 2), we retained 19 references for this systematic review.

**Table 1:** Number of references extracted from each database.

Database	Query result, n
ACM^a^	584
IEEE Xplore	12
ProQuest	1678
Scopus	44
Web of Science	16
ERIC^b^	2
Springer	1952
Google Scholar	100

^a^ACM: Association for Computing Machinery.

^b^ERIC: Education Resources Information Center.

## Results

### Overview

The 19 included articles were published between 2013 and 2021. Their number per year increased from 1 in 2013 to 2 in 2017 and up to 5 in 2018. Subsequently, the number of publications decreased to a mean of 3 per year.

The selected articles were published in conferences and journals focused on five different themes (Table 2): medical and public health, medical and health informatics, human-computer interactions, physical human behavior, and engineering and science. The three most popular themes were medical and health informatics, human-computer interactions, and engineering and science (15/19, 79%). Among the included articles, four were published in JMIR publications: three in JMIR mHealth and uHealth and one in JMIR Public Health and Surveillance.

**Table 2:** Conference proceedings and journals in which the included articles were published (N=19).

Conference or journal		Reference
**Medical and public health**		
	BMJ Open	Aguilera et al [7]
	Public Health Nutrition	Lee et al [8]
**Medical and health informatics**		
	JMIR mHealth and uHealth	Zhou et al [9], Rabbi et al [10], Galy et al [11]
	JMIR Public Health and Surveillance	Fukuoka et al [12]
	Journal of Biomedical Informatics	Sprint et al [13]
**Human-computer interactions**		
	Proceedings of the ACM on Human-Computer Interaction	Zhu et al [14]
	User Modeling and User-Adapted Interaction	Gasparetti et al [15]
	Journal of Ambient Intelligence and Humanized Computing	Batool et al [16]
	Multimedia Tools and Applications	Angelides et al [17]
	Adjunct Publication of the 26th Conference on User Modeling, Adaptation and Personalization	Schäfer et al [18]
**Physical human behavior**		
	Journal of Behavioral Medicine	Forman et al [19]
	Journal of Electromyography and Kinesiology	Hermens et al [20]
**Engineering and science**		
	Applied Sciences	Chen et al [21]
	Sensors	Dijkhuis et al [22]
	Springer Proceedings in Complexity	Mollee et al [23]
	IEEE Access	Diaz et al [24]
	International Conference on Industrial, Engineering and Other Applications of Applied Intelligent Systems	Mollee and Klein [25]

### Sensor Types and Data Capture

The characteristics of the sensors (eg, number and type) used to capture PA behaviors and of the collected raw data are summarized in Table 3.

The length of data recordings varied between 4 days and 1 year, with a median of 70 days. Recording lasted ≤7 days in two studies, between 3 and 5 weeks in six studies, between 10 and 16 weeks in eight studies, and ≥6 months in three studies.

The number of participants varied between 10 and 11,615, with *<*30 in five studies, between 30 and 299 in 10 studies, and ≥300 participants in four studies.

All included studies used accelerometer sensors. We could categorize these devices into three groups: (1) commercial wrist-worn wearable accelerometers that are consumer-grade devices with a sample rate between 30 Hz and 60 Hz, such as Fitbits [13,14,19,22,25], Samsung Gear [17], and Nokia [15]; (2) smartphone accelerometers with a sample rate usually set to 50 Hz and up to 100 Hz, in which data were collected via an app installed in the smartphone [7,9,10,16,18]; and (3) scientifically validated wearable accelerometers with a sample rate up to 100 Hz, such as ActiGraph [8], GENEActiv [11,24], and other devices developed for health care [12,20].

In 7 out of the 19 (37%) selected studies, accelerometers were used with other sensors such as GPS tracking [10,16,17], compass position tracking [17,20], heart rate trackers [17,21], and smart scales [15,19].

The recorded raw data varied in function of the sensor characteristics, including sampling frequency, accuracy, and axis number. Moreover, other sensor features such as battery duration and storage capacity affected the recording length. For instance, a long battery life and high storage capacity enable longer recording without interruptions. Table 4 summarizes the number of participants and data recording duration for the included studies.

**Table 3:** Number of sensors, device type and model used, and raw data generated.

Sensor type	Device and model	Raw data	Reference
Accelerometer	ActiGraph GT1M uniaxial	Uniaxial accelerometry	Lee et al [8]
Accelerometer	GENEActiv triaxial accelerometer	Gravity-subtracted signal vector magnitudes (SVMgs) per second	Galy et al [11], Diaz et al [24]
Accelerometer	Generic device from the mobile phone	Acceleration (sample rate not specified)	Aguilera et al [7], Zhou et al [9]
Accelerometer	Triaxial accelerometer (HJA-350IT, Active Style Pro, Omron Healthcare Co, Ltd)	Triaxial acceleration (6 Hz)	Fukuoka et al [12]
Accelerometer	Fitbit	Triaxial acceleration (sample rate not specified)	Zhu et al [14]
Accelerometer	Fitbit Flex	Triaxial acceleration (sample rate not specified)	Dijkhuis et al [22]
Accelerometer	Fitbit Charge HR and Fitbit Flex	Triaxial acceleration (sample rate not specified)	Sprint et al [13]
Accelerometer	Fitbit One	Triaxial acceleration (sample rate not specified)	Mollee and Klein [25]
Accelerometer	Not specified	Not specified	Mollee et al [23]
Accelerometer	Smartphone and Actigraph (GT3X model)	Triaxial acceleration (sample rate not specified)	Schäfer et al [18]
Accelerometer and heart rate monitor	Mix of devices and models	Accelerometry, heart rate monitor, PA^a^ information, and user information (sample rate not specified)	Chen et al [21]
Accelerometer, GPS, self-log PA, and food	Smartphone	Smartphone accelerometry, GPS data, PA and food logs with sample rate specified	Rabbi et al [10]
Activity tracker, smart scale, and smartphone (what they ate and drank in the Fitbit app)	Fitbit Flex 2 activity tracker, Yunmai smart scale, smartphone	Accelerometry, weight and food logs (sample rate not specified)	Forman et al [19]
Accelerometer, gyroscope, and magnetic compass	ProMove-3D (developed by Inertia Technology)	Accelerometry (sample rate not specified)	Hermens et al [20]
Accelerometer and GPS	Smartphone	Accelerometry and GPS (sample rate not specified)	Batool et al [16]
Triaxial accelerometer, heart rate monitor, GPS, 3-axis gyroscope, digital compass, altimeter, light sensor	Samsung Gear Fit and Fitbit Surge	Accelerometry, heart rate data, GPS, 3-axis gyroscopes, digital compass, altimeter, light sensor (sample rate not specified)	Angelides et al [17]
Accelerometer, heart rate, and smart scale	Nokia; models not specified	Accelerometer, heart rate data, and smart scale (sample rate not specified)	Gasparetti et al [15]

^a^PA: physical activity.

**Table 4:** Length of data recording and number of participants among the included studies.

Length of recording			Participants, n		Reference
**1 to 7 days**					
	4 days	1714		Lee et al [8]	
	7 days	215 (women)		Fukuoka et al [12]	
**1 to 5 weeks**					
	3 weeks	17		Rabbi et al [10]	
	3 weeks	48		Zhu et al [14]	
	1 month	14		Angelides et al [17]	
	4 weeks	24 (adolescents)		Galy et al [11]	
	4 weeks	74 (children)		Schäfer et al [18]	
	5 weeks	87 (children)		Diaz et al [24]	
**6 to 20 weeks**					
	10 weeks	11		Schäfer et al [18]	
	10 weeks	64		Zhou et al [9]	
	3 months	10		Hermens et al [20]	
	12 weeks	48		Dijkhuis et al [22]	
	12 weeks	108		Mollee and Klein [25]	
	3 months	269		Chen et al [21]	
	12 weeks	2472		Mollee et al [23]	
	16 weeks	52		Forman et al [19]	
**21 weeks to 1 year**					
	6 months	276		Aguilera et al [7]	
	6 months	500		Batool et al [16]	
	1 year	11,615		Gasparetti et al [15]	

### Data Preprocessing

Raw data extracted from sensors need to be transformed into variables that will contribute to generating the input features for data mining models to detect PA behavior changes. Table 5 provides a summary of the initial transformation and the resulting preprocessed data.

The preprocessing of the raw data from sensors was carried out in two ways. The first approach was to use proprietary programs to transform the sensors’ data directly into the resulting preprocessed data, without specifying whether there was an initial preprocessing stage such as that used to generate steps, metabolic equivalents (METs), calories, heart rate, or exercise characteristics (type, duration, distance, or frequency). The second approach was to produce intermediate data that were then transformed in the resulting preprocessed data using a custom preprocessing tool. For instance, to generate PA levels (PALs), raw data were first transformed into MET, activity classes, or signal vector magnitudes.

The resulting preprocessed data were mainly activity characteristics (step count, PAL, integrals of the moduli of acceleration, activity types, duration, distance travelled, and frequency) and energy expenditure (MET and calories). Step count from smartphones and commercial wrist-worn devices was the most frequent, followed by PAL from research-grade devices.

The resulting preprocessed data were aggregated at different time levels (Table 6). Day and minutes were the most frequent time levels of aggregation. Generally, PAL and MET were aggregated per minute. Calories and step counts were calculated per day.

**Table 5:** Summary of data preprocessing variables.

Resulting/initial preprocessing			Reference
Steps: unknown (proprietary program)			[7,9,13-15,17,19,22,25]
Metabolic equivalents: unknown (proprietary program)			[21]
Calories: unknown (proprietary program)			[10,17,19]
Exercise characteristics: unknown (proprietary program)^a^			[10,17,21]
Sleeping time: unknown (proprietary program)			[15,17]
Weight: unknown (proprietary program)			[15,19]
Heart rate: unknown (proprietary program)			[17,21]
**Physical activity (PA) levels**			
	Signal vector magnitudes	[11,24]	
	PA counts	[8]	
	Metabolic equivalents	[12]	
	Activity classes	[18]	
	Not specified	[23]	
Integrals of the moduli of acceleration signals			[20]
Actual activity level; not specified^b^			[16]

^a^Type, duration, distance, frequency.

^b^Definition of activity level was not specified.

**Table 6:** Aggregation level of the resulting preprocessed data.

Reference		Month	Week	Day	Hour	Minute	Seconds	Not specified
Angelides et al [17]		✓	✓	✓	✓			
Zhou et al [9]				✓				
Aguilera et al [7]				✓				
Zhu et al [14]				✓				
Mollee and Klein [25]				✓				
Forman et al [19]				✓				
Gasparetti et al [15]				✓				
Chen et al [21]		✓				✓		
Dijkhuis et al [22]					✓			
Lee et al [8]						✓		
Fukuoka et al [12]						✓		
Sprint et al [13]						✓		
Schäfer et al [18]						✓		
Diaz et al [24]							✓	
Galy et al [11]							✓	
Mollee et al [23]								✓
Hermens et al [20]								✓
Rabbi et al [10]								✓
Batool et al [16]								✓

### Features Used to Detect and Extract Behavior Changes

The features of the data mining models were mostly generated from the sensors’ preprocessed data and, in some cases, from other sources (nonsensor data). Table 7 provides the features categorized with respect to the function of their source: accelerometers, other sensors, and nonsensor devices.

Most of the included articles used descriptive statistics to present the preprocessed data as features, for instance total number of steps per day [9,11,14,17,25], mean number of steps per day [17], or PA count per hour [8]. Other studies created windows or segments of time to calculate PA characteristics, including segments of steps or sleep [15] and PA bouts [13,24]. Other articles used the preprocessed data to calculate the participants’ step achievements such as whether they reached their step goal [9,11,19,23]. Zhu et al [13] used more complex features such as the ratio between the most active and least active period or the circadian rhythm strength.

In addition to the features derived from sensors, others were created from measurements carried out during the intervention by scientists, such as the number of days that a person participated in the intervention [19] and anthropometric [7,21] or psychological [14,16,25] characteristics. Data were collected through surveys/questionnaires or interviews with participants.

**Table 7:** Features used for data mining to detect behavior changes.

Reference	Features derived from accelerometers	Features derived from other sensors	Features derived from nonsensor devices
Aguilera et al [7]	Number of minutes of activity in the last day, cumulative number of minutes of activity this week, fraction of activity goal, fraction versus expected activity goal at this point in the week	Number of days since each feedback message was sent	Age, gender, language, 8-item Patient Health Questionnaire (depression) score
Hermens et al [20]	Not specified	Not specified	Not specified
Chen et al [21]	Monthly mean metabolic equivalent of task, effective exercise time, type, frequency	Monthly mean exercise and resting heart rate	Gender, height, weight, age
Forman et al [19]	Days where PA^a^ goal is met	Sum of days with self-monitored weight, days with self-monitored eating, days where calorie goal is met, weight loss in pounds	Number of days in the intervention period
Gasparetti et al [15]	Consecutive daily segments of steps, consecutive daily segments of sleep	—^b^	—
Batool et al [16]	Actual activity level	—	Desired activity level, intention (attitude, subjective norms, perceived behavioral control), habit, and 16 demographic features (eg, age, gender, marital status)
Dijkhuis et al [22]	Hour of the workday, number of steps for that hour, number of steps in the past hour, total number of steps up to that hour, mean number of steps of workdays	—	—
Rabbi et al [10]	PA frequency and calories	—	—
Zhou et al [9]	Daily steps and goal	—	—
Angelides et al [17]	Total and mean hourly, daily, weekly, and monthly sleep duration; sleep calories; exercise duration; exercise distance; exercise calories; step count; step distance; step calories; BMI; and basal metabolic rate	—	Height (cm), weight (kg), age, gender
Diaz et al [24]	Hourly and daily frequency, and mean time spent in moderate to vigorous PA bouts of at least 3, 10, and 30 seconds, and in sedentary bouts of at least 60, 120, and 300 seconds	—	—
Galy et al [11]	Total daily time spent in light/moderate/vigorous PA, total daily number of steps, and a binary goal achievement feature	—	—
Fukuoka et al [12]	Mean metabolic equivalent of tasks per minute, mean moderate-to-vigorous PA per minute	—	—
Lee et al [8]	24-hour mean PA count on weekdays and 24-hour mean PA count on weekends	—	—
Sprint et al [13]	Steps, PAL^c^ and bouts count, mean, percentages, ratios and SD. Circadian rhythm time-series statistics and texture features from an image-processing technique	—	—
Mollee et al [23]	Impact of online community (sharing my PAL with peers), target PAL and goal achievement	—	—
Schäfer [18]	PAL per minute	—	—
Mollee and Klein [25]	Daily steps	—	Psychological questionnaire scores for self-efficacy, barriers, social norm, long-term goals, intentions, satisfaction, outcome expectations
Zhu et al [14]	Daily steps	Motivation to exercise (Likert scale)	Iowa-Netherlands Comparison Orientation Measure-23 (INCOM-23) for social comparison (psychometrics)

^a^PA: physical activity.

^b^Not applicable.

^c^PAL: physical activity level.

### Data Mining

#### Algorithm Overview

Table 8 summarizes the data mining methods and specific algorithms used in the selected articles.

Clustering was the most used method, particularly the K-means algorithm. Indeed, in health interventions, the PA performed by each participant varies in duration, form, and intensity. Therefore, an algorithm that clusters PA behaviors is required to analyze them. The unsupervised K-means algorithm is suitable for this task. Indeed, due to its simplicity and ease of use, this is one of the most popular options for data mining [26]. Decision-making algorithms and classifiers were the second most used methods. Both rely on supervised algorithms that use PA characteristics as a method for predicting when and/or what information must be delivered to individual participants for increasing their PA. Other algorithms were also tested to extract PA behaviors, such as social cognitive and contagion models, PA windows permutations, and recommendation algorithms.

**Table 8:** Data mining methods and algorithms.

Data mining method and algorithm			Reference
**Classifiers**			
	K-nearest neighbor and support vector machine	[20]	
	Random forest	[22]	
	Random forest and weighted score	[18]	
	Shallow neural networks	[16]	
**Clustering techniques**			
	K-means	[8,11,12,24]	
	Agglomerative	[21]	
	Partitioning around medoids and reinforcement learning	[15]	
**Decision-making algorithms**			
	Multiarmed bandit	[10]	
	Multiarmed bandit upper confidence bound	[19]	
	Reinforcement learning multiarmed bandit	[7,9]	
	Behavioral analytics algorithm		
	MAB^a^	[14]	
Social cognitive model for predicting exercise behavior change			[25]
Social contagion model combined with a linear model			[23]
Physical activity change detection: small window permutation-based change detection in activity routine			[13]
Recommendation: genetic algorithms and Pareto optimality			[17]

^a^MAB: multiarmed bandit.

#### Classifiers

Hermens et al [20] used a k-nearest neighbor model and a support vector machine to determine whether a specific time of the day was suitable for sending a motivational message to optimize adherence to the intervention. Dijkhuis et al [22] used a tree and tree-based ensemble algorithm classifiers to predict whether users will achieve their daily PA goal. On the basis of this prediction, a personalized PA coaching program was proposed. Forman et al [18] developed gamified personalized feedback using a score model depending on the PA change detected from accelerometer data. Batool et al [16] predicted the likelihood that the PA level of a given patient was too low. They also predicted which patients were at higher risk of not adhering to the prescribed therapy to optimize their PA.

#### Clustering Techniques

Lee et al [8] grouped participants in two clusters on the basis of their step counts (one more active than the other), and analyzed them to better understand these PA patterns. Diaz et al [24] used a clustering-based approach for a more insightful analysis of the participants’ PA behavior and of the nature of the PA behavior changes, if present. Galy et al [11] clustered PA levels and daily step goal achievement to assess the adherence to a health program. Fukuoka et al [12] identified PA clusters to analyze and compare sociodemographic features and cardiometabolic risks among participants belonging to these clusters. Chen et al [21] clustered the participants’ PA, and then established a system to adapt the exercise program for the next week as a function of the individual PA behavior change. Gasparetti et al [15] clustered the participants’ PA to generate groups of habits recommended by a system to the participants with the objective of changing their PA to obtain weight loss effects.

#### Decision-Making Algorithms

Rabbi et al [10] generated personalized suggestions in which users were asked to continue, avoid, or make small changes to their existing PA behaviors in order to help them reach their PA goals. Forman et al [19] developed an algorithm that could personalize and optimize the PAL during the intervention as a function of the amount of PA performed. Aguilera et al [7] generated personalized messages for participants in the intervention to increase their PA and consequently the intervention effectiveness. Zhou et al [9] adapted the step goal settings of the intervention depending on the PA behavior change. Zhu et al [14] personalized social comparison among participants to motivate them toward improving their PA behavior.

#### Social Cognitive Model

Mollee and Klein [25] developed a model that simulates changes in PALs over 2 to 12 weeks to optimize the participants’ health outcome.

#### Social Contagion Model

Mollee et al [23] used a social contagion model to explain the PAL dynamics in a community.

#### PA Windows Permutations

Sprint et al [13] proposed a window-based algorithm to detect changes in segments of users’ PA behavior to motivate progress toward their goals.

#### Recommendation Algorithms

Angelides et al [17] used genetic algorithms and Pareto optimality to compare the participants’ and peer community’s data to help participants interpret the PA data and to generate personal lifestyle improvement recommendations.

### Interpretation of the Data Mining Models

#### Overview of Models

The resulting data mining models detecting PA behavior changes were used for several purposes, as summarized in Table 9 and below.

**Table 9:** Main uses of the resulting data mining models.

Main use	Reference
Personalized feedback	[7,10,15,16,18,20]
Personalized program	[9,19,21,22]
Support for self-reflection	[17]
Cohort analysis of the intervention impact on PA^a^	[8,11-13,24]
Analysis of the social component effects on PA	[14,23,25]

^a^PA: physical activity.

#### Personalized Feedback

The PA behavior changes extracted from participants’ data were used to promote PA by creating and sending personalized messages that reported the behaviors and gave suggestions for achieving the previously established PA goals. For instance, Aguilera et al [7] built a system that detects the participants’ PA behavior changes and generates personalized daily text messages with custom timing, frequency, and feedback about their step count/goal and motivational content. Hermens et al [20] built a system that chooses the best suitable time to send a message with personalized intention, content, and representation. Schäfer et al [18] created an app with gamified feedback where different avatars are awarded based on the participant’s daily PA behavior. Gasparetti et al [15] suggested personalized PA patterns based on the participants’ PA patterns. Batool et al [16] detected the participants’ PA behavior while commuting and suggested how to increase it. Rabbi et al [10] generated personalized simple PA suggestions (continue, avoid, or make small changes).

#### Personalized Programs

The PA intervention program and objectives are adapted to each participant’s needs. For instance, Chen et al [21] created a guided exercise prescription system that adapts as the participants’ PA behavior changes. Similarly, Forman et al [19] changed the participant’s exercise intensity suggestion depending on their PA behavior achievements. On the basis of each participant’s step count progress, Dijkhuis et al [22] suggested new daily step objectives. Zhou et al [9] used push notifications to deliver daily step goals.

#### Support for Self-Reflection

Algorithms can help participants to interpret their PA behavior changes. For example, Angelides et al [17] used an algorithm to assist in the interpretation of the participant’s PA data by comparing them with those of the peer community and to generate personalized recommendations to achieve their daily goals.

#### Cohort Analysis of the Intervention Impact on PA

These algorithms detect PA behavior changes in participants that allow analyzing the intervention impact. For example, Fukuoka et al [12] determined PA patterns in women throughout the day that could help to develop more personalized interventions and guidelines. Diaz et al [24] analyzed the changes in PA behavior (bouts and frequency) during an intervention. Galy et al [11] tracked the participants’ adherence to the international recommendations during an intervention. Lee et al [8] identified PA patterns associated with specific subgroups of people who participated in an intervention. Sprint et al [13] analyzed the participants’ PA changes during an intervention by comparing multiple time windows.

#### Analysis of the Social Component Effect on PA

These algorithms analyze the psychosocial influences on the participants’ PA. For example, Mollee et al [23] analyzed the PA dynamics in a community using a social contagion model. Mollee and Klein [25] analyzed the PA dynamics in a networked community using social cognitive theories, and Zhu et al [14] personalized social comparison during an intervention to increase the participants’ PA.

The main uses can be classified in two groups. The first group, composed of 11 out of the 19 (58%) selected studies, aimed to generate personalized feedback/PA programs to scaffold and support PA behavior changes among participants. Indeed, researchers seem inclined to generate greater personalization because it increases the intervention efficiency, effectiveness, enjoyment, and reliability [27]. The second group, composed of 8 out of the 19 (42%) selected studies, sought to analyze the impact of interventions on the participants’ PA. Specifically, these studies analyzed the intervention impact on PA at the cohort level to assess health education interventions, and analyzed participants’ PA to show them their behaviors and help to understand them. The main objective of both groups was to explore how PA behavior patterns relate to the intervention effectiveness, which can add new evidence on how to create more effective interventions [28].

## Discussion

### Principal Findings

#### Summary

We found 19 articles about data mining models and techniques to detect PA behavior changes in health education or promotion studies, and their number has progressively increased over time. We here discuss the principal findings, identify opportunities and challenges for future research directions, and present the limitations of this systematic review. The Discussion is structured according to the RQs as a guide.

#### Opportunities and Challenges

##### Sensor Types and Data Capture

All selected studies used accelerometer sensors to capture PA behaviors. While 7 out of the 19 (37%) studies utilized accelerometers exclusively, the rest employed them with other sensors. Nonaccelerometer sensors capture additional information that may be relevant to PA (such as work/school schedule, itineraries, and sleep patterns [29]) and could yield auxiliary features for the data mining models. For instance, GPS sensors provide the number of kilometers and location of PA performed.

The median number of participants in the selected studies was 74, and participants were mainly young or middle-aged adults. This low number of participants and the skew toward adults may have generated biased data mining models that can detect and find behavior changes only in a specific population. Different population groups behave differently and should be studied independently. For instance, PA behaviors are different in children and adults [2]. Some of the studies focused on groups with specific PA behaviors, such as children [18,24], adolescents [11], and women [12]. However, some population groups with distinctive PA patterns, such as pregnant women [30] and people with health conditions or disabilities [31], may need custom detection models.

In 15 out of the 19 (79%) included studies, data were recorded for less than 3 months. Therefore, the current methods for detecting PA behavior changes have been developed mostly for capturing short-term patterns, making the conclusions valid only for short periods. To detect medium- and long-term PA behavior changes, studies with more extended recording periods are needed, such as the study by Gasparetti et al [15] based on data collected during 1 year. Moreover, new methods to detect extended (eg, annual or seasonal) PA patterns are required to study how the participants’ behavior and habits change over time. An increase in the participants’ number and recording length will lead to new challenges related to big data analysis, such as efficient data management and data mining processing speeds.

##### Data Preprocessing

Many of the selected studies used commercial accelerometers that allow only the retrieval of aggregated preprocessed data using proprietary software (ie, number of steps per minute), without being transparent on how data were preprocessed (ie, how steps were calculated from the accelerometry data). This data preprocessing black box makes it impossible to determine the quality of the captured PA data and makes the data mining results scientifically irreproducible. Conversely, in studies that used medical-grade accelerometers, the accelerometry data were explained in detail and the preprocessing steps were documented and referenced.

We found a lack of standard procedures for data preprocessing that made it challenging to compare the study results and conclusions. Indeed, if data are not preprocessed correctly, this could cause the transfer of incorrect information to the features and then to the data mining models. This could lead to the creation of inaccurate models, thus limiting the study validity. Data cleaning is a good example of this issue. Indeed, the best procedure to eliminate the nonwearing time remains unclear along with the impact on the accuracy of the resulting models. If nonwearing time is poorly removed, features can generate a PA underestimation by recognizing nonwearing time as sedentary behavior when it is not. Moreover, if sensor data concerning changes in accelerations while commuting by car or bus are not completely removed, they will be erroneously classified as steps, thereby overestimating PA in the model and in the conclusions. Similarly, sedentary activities could be overestimated if sleep time is not correctly removed.

Most of the selected studies aggregated information by day or minute. Although data aggregation is useful when comparing general features of PA behaviors, such as daily steps, this procedure may overlook subtle behavioral changes that can be crucial for detecting major PA behavior changes. For instance, if a person who walks every morning decides to change their behavior and starts to walk at night, the sum of daily steps will be the same, but this new behavior will not be detected. Conversely, it could be detected if the aggregation level is changed to the hour. To detect these and other subtle behavior changes, PA should be analyzed simultaneously at different aggregation levels, and new time frames should be created to match daily habits and behaviors, such as periods of the day (eg, morning, afternoon) or participants’ office hours.

##### Features Used to Detect and Extract Behavior Changes

Most of the preprocessed data were transformed into features that are simple descriptive statistics, such as the total time spent at a specific PAL or the mean number of steps. These features are valuable to detect behavior changes, but they mainly capture the PA intensity and the PA presence or absence. Yet, PA has more valuable characteristics that vary during PA behavior changes and that can help to detect such behavior changes, such as the length of PAL bouts or the amount of time spent doing PA. These PA characteristics can be extracted from current sensor data. For instance, Galy et al [11] explored different moderate-to-vigorous PA bout lengths and Sprint et al [13] assessed the circadian rhythm. International PA guidelines can serve as inspiration to identify new PA features. For instance, according to World Health Organization recommendations, adults should perform muscle-strengthening activities (involving all major muscle groups) at moderate or higher intensity at least twice per week [2]. This calls for the creation of features that capture the muscle activity type, intensity, and frequency. Moreover, most of the included studies used only PA-derived features to detect behavior changes, and did not consider relevant non-PA data associated with PA changes, such as the participants’ weight and quality of sleep. Some studies captured non-PA data, but they did not use them to detect PA changes. For instance, Rabbi et al [10] used only PA-derived data (PA frequency and calories burned) to detect behavior changes, although they also recorded the participants’ food intake, thus excluding their caloric intake that is closely related to weight and the amount of PA participants are likely perform.

The use of simple descriptive statistics as features and the exclusion of non-PA data associated with behavior changes indicate that sensor data were underexploited and that the features used to detect PA behavior changes are still underdeveloped. Including new PA characteristics and new non-PA features could help to better understand the nature of PA changes and how these features influence PA behavior changes, ultimately increasing the model detection accuracy.

##### Data Mining Methods and Techniques

Most studies used off-the-shelf classifiers, clusters, and decision-making algorithms to detect PA behavior changes. We expected to find tailor-made algorithms because in health education settings, it is important to find specific PA patterns in participants of different classes who follow learning modules with different contents and with different PA goals. Moreover, we noticed that most authors did not explain how they chose the algorithms and did not specify the efficiency and accuracy of the models used for detecting PA behavior changes, raising uncertainty about how good they are at this task. This suggests that more efficient and accurate algorithms could be created and calls for more transparency in the algorithm choice process. Therefore, authors should explicitly describe the steps and methodology of new algorithms, and share their source codes to be scrutinized and to compare their detection accuracy. The creation of open accelerometry databases is also needed to enable benchmarking.

##### Interpretation of the Resulting Data Mining Models

The main uses of the data mining models focused on personalization, support for self-reflection, and analysis of PA behaviors. Model interpretation focused on generating personalization and support for promoting behavior changes. Personalized feedback and intervention programs were based mostly on the participants’ PA data. The inclusion of additional information that may influence behavior changes (eg, contexts, schedules, social constraints, motivation, and weather) would allow for better interpretation and use of the detected behavior changes. Systems could exploit these additional data to improve the feedback delivery time and content, with positive effects on the effectiveness of health education programs and interventions. For instance, with the current models, a participant could receive an automatized personalized behavior change message that suggests taking a short walk, although it is snowing outside. This would decrease the likelihood of following the suggestion. However, if the system could be aware of the weather, the participant would receive this suggestion only after the weather conditions have improved, or a different suggestion that is more likely to trigger a behavior change at that point in time. Moreover, as the models relied mainly on PA features to model and interpret the behavior changes, only the physical dimension of the learning process in health education was incorporated in the models and their interpretation, leaving aside the knowledge dimension of the learning process. Learning management systems and intelligent tutoring systems already capture the knowledge dimension. Their integration would help to understand, in a comprehensive way, how participants learn, and would enable the real-time monitoring of how PA behavior changes align with the intervention purpose. This would allow adapting each participant’s content and learning objectives in real time, thereby improving instructions and learning, ultimately increasing the program or intervention effectiveness.

Most of the included studies generated complex output models that require detailed knowledge of how they were created to interpret the resulting patterns, making them difficult to understand for health scientists and any other scientist not familiar with machine learning. This is a common problem in interdisciplinary teams; however, an effort can be made to create more readable, intuitive, and easy-to-understand algorithms and methods, a goal that exists in related machine learning areas such as explainable artificial intelligence [32].

### Limitations

Studies on wearable machine learning devices to detect changes in PA in health education have only started to be published in the last decade. As research is advancing, keywords are changing and new terms are created. Although we used a wide range of keywords in our query to include sensors, PA, and health education, we may have left some keywords out, and thus we may have missed some references. This may have also affected the initial reference screening process by title and abstract. We minimized this issue by testing several queries before starting our systematic review until we found the one we ultimately used. Another possible limitation in our search is that we might have omitted references listed only in other peer-reviewed databases (we searched only the most popular databases in engineering and computer science), such as medical databases (ie, PubMed). We mitigated this risk by including grey literature in our systematic review (see the Methods section).

Regarding the research subquestions and the review structure, we created research subquestions in line with the usual data mining process steps, but we certainly left some topics unaddressed. For instance, we did not address ethics, privacy, and security issues, or how data are filtered during preprocessing (eg, sleeping time or sensor nonuse). Although these are common substeps during the data mining process and including them would have made this systematic review more comprehensive, we preferred to limit this review only to the critical steps.

### Conclusions

In the last 10 years, different methods have been developed to detect behavior changes in health education or health promotion contexts. These methods have been tested in small populations, are based on short data-recording periods, and rely mainly on accelerometry data. Incorporating information that is complementary to the participants’ PA data would allow for creating more precise detection models, better interpreting these models, and understanding how participants learn and what triggers new behaviors. Exploring other data aggregation levels, in addition to days and minutes, could help to detect more subtle and long-term behavior changes. Fully describing the data preprocessing methods and the efficiency and accuracy of the behavior change detection models would help to better understand, scrutinize, and compare studies. Detection models were mainly used to generate personalized feedback and to provide support for promoting or maintaining behavior changes, but did not integrate the knowledge dimension of the learning process. Adding the knowledge dimension and creating easier-to-understand models could facilitate the interpretation of participants’ behavior changes in a more comprehensive way, opening the way toward better and deeper analyses and personalization.

## Abbreviations

ACM: Association for Computing Machinery
ERIC: Education Resources Information Center
MET: metabolic equivalent
PA: physical activity
PAL: physical activity level
PRISMA: Preferred Reporting Items for Systematic Reviews and Meta-Analyses
RQ: research question
